# Data of knowledge towards Zika Virus infection in Sabah, Malaysia

**DOI:** 10.1016/j.dib.2022.108006

**Published:** 2022-03-01

**Authors:** Noor Ain Haron, Syed Sharizman Syed Abdul Rahim, Jaeyres Jani, Nur Athirah Yusof, Zarina Amin, Hooi Yuen Khoo, Hai Yen Lee, Chee Sieng Khor, Kim Kee Tan, Mohd Rohaizat Hassan, Chin Mun Wong, Hani Kartini Agustar, Adia Aqilla Samsusah, Rozita Hod, Sazaly Abu Bakar

**Affiliations:** a Biotechnology Research Institute, Universiti Malaysia Sabah, Sabah 88400, Malaysia; b Department of Public Health Medicine, Faculty of Medicine and Health Sciences, Universiti Malaysia Sabah, Sabah 88400, Malaysia; c Borneo Medical and Health Research Centre, Faculty of Medicine and Health Sciences, Universiti Malaysia Sabah, Sabah 88400, Malaysia; d Tropical Infectious Diseases Research and Education Centre (TIDREC), Higher Institution Centre of Excellence (HICoE), Universiti Malaya, Kuala Lumpur 50603, Malaysia; e Department of Community Health, Faculty of Medicine, Universiti Kebangsaan Malaysia, Kuala Lumpur 56000, Malaysia; f Department of Earth Sciences and Environment, Faculty of Science and Technology, Universiti Kebangsaan Malaysia, Selangor 43600, Malaysia

**Keywords:** Zika, Community perceptions, Zika in Sabah, Knowledge on Zika

## Abstract

This dataset presents a cross-sectional survey and was conducted to assess the knowledge on Zika Virus infection among adults in Sabah. The data were collected from December 2019 to February 2021, 274 adults living in forest fringe communities were interviewed by trained personnel and have completed the distributed questionnaires. SPSS version 27.0 was used to analyzed the data. These data could serve as auxiliary information and/or research data for other researchers in Sabah. It could also serve as guide or reference data to other researchers outside Sabah who may be interested in carrying out similar research in other state.

## Specifications Table

**Table:** 

Subject	Epidemiology
Specific subject area	Health and medical sciences
Type of data	Primary data Tables Survey data collected via questionnaire
How the data were acquired	Data were collected via validated questionnaire through face-to-face interview by trained personnel. It was then analysed using SPSS and R.
Data format	Raw, analyzed
Description of data collection	This was a cross sectional study that was performed from December 2019 to February 2021. There was slight delay in data collection due to movement restriction during the COVID-19 pandemic. There were 274 respondents interviewed via multistage sampling. The process includes: 1. All participants have been provided informed consent and will be analyzed anonymized. 2. A socio-demographic profile of survey participants will be carried out in our study which includes Sabah resident population with age ranging from 18 to 60 years old. 3. The survey fieldwork were carried out five sites geographically distributed around Sabah.
Data source location	Region: Southeast Asia Country: Malaysia State: Sabah Districts: Pulau Gaya, Ranau, Sandakan, Telipok and Likas
Data accessibility	Repository name: Mendeley data Data identification number: 10.17632/c33wtk56v6.1 Direct URL to data: https://data.mendeley.com/datasets/c33wtk56v6/1

## Value of the Data

• The data provide important baseline information on knowledge towards Zika Virus infection among forest fringe and rural residential area in Sabah. All researchers can benefit from these data to help in preventing Zika Virus infection and support for health education to increase their awareness.
• The data are useful to explore the level of understanding among adults living in rural areas, therefore able to tailor prevention strategies to suit local needs and understanding.
• The data will become an important source to understand the relationship between Zika risk of infection and community perspective.

## 1 Data Description

This was a cross sectional study that was performed from December 2019 to February 2021. There was a slight delay in data collection due to movement restriction during the COVID-19 pandemic. The questionnaires were adapted from World Health Organization [1] and incorporates demographic, comorbidity, socioeconomic, accessibility and knowledge on Zika infection. To validate, the questionnaire was thoroughly reviewed by the Public Health Specialists to ensure the questions were not ambiguous and content was appropriate. There were 274 respondents interviewed and all the responses content in the questionnaires were deemed valid for statistical analysis. This data was analyzed with SPSS 27.0 using a common scoring method where a score of 1 will be given for each correct answer and 0 for each wrong or no answer [2].

The analysis of sociodemographic factors is given in Table 1. Majority of them were from the district of Sandakan (*n* = 100, 36.5%). Adults of Bajau ethnicity (*n* = 107, 39.1%) and females (*n* = 196, 71.5%) made up the majority of the study participants. Age ranging from 18 years old to 40 years old were the highest (*n* = 132, 48.2%) and mostly with household income less than RM3000 (*n* = 264, 96.4%). More than half (*n* = 195, 71.1%) have attained at least a primary school education.

**Table 1:** Analysis of sociodemographic factors.

Variables	Awareness *n* (%)	No Awareness *n* (%)	Crude Odds ratio (cOR)	95% C.I	χ2	*p* value
**Age**						
18–40	58 (43.9)	74 (56.1)			2.842	0.24
41–60	53 (44.5)	66 (55.5)				
61 and above	6 (26.1)	17 (73.9)				
**Gender**						
Male	29 (37.2)	49 (62.8)	0.726	0.42–1.25	1.359	0.24
Female	88 (44.9)	108 (55.1)				
**Place**						
Sandakan	44 (44.0)	56 (56.0)			58.968	<0.001*
Likas	12 (15.2)	67 (84.8)				
Pulau Gaya	19 (44.2)	24 (55.8)				
Telipok	26 (92.9)	2 (7.1)				
Ranau	16 (66.7)	8 (33.3)				
**Marital status**						
Single	29 (49.2)	30 (50.8)	1.395	0.78–2.49	1.279	0.26
Married	88 (40.9)	127 (59.1)				
**Ethnicity**						
Bajau	23 (21.5)	84 (78.5)			55.468	<0.001*
Sungai	29 (58.0)	21 (42.0)				
Dusun	31 (75.6)	10 (24.4)				
Kadazan	2 (100)	0 (0)				
Melayu	7 (100)	0 (0)				
Others	25 (37.3)	42 (62.7)				
**Other comorbid**						
Yes	24 (51.1)	23 (48.9)	0.665	0.35–1.25	1.622	0.20
No	93 (41.0)	134 (59.0)				
**Education**						
None	5 (6.3)	74 (93.7)			82.447	<0.001*
Primary	32 (41.0)	46 (59.0)				
Secondary	59 (62.1)	36 (37.9)				
Tertiary	21 (95.5)	1 (4.5)				
**Households’ income per month**						
<RM 3000	107 (40.5)	157 (59.5)	0.405	0.35–0.469	13.927	<0.001*
≥RM 3000	10 (100)	0 (0)				

⁎ *p*-value < 0.05.

## 2 Experimental Design, Materials and Methods

To reiterate, this research is done to evaluate the knowledge gaps among the forest fringe residents towards Zika Virus. It was done via multistage random sampling [3] by selecting five districts in Sabah. From the five districts that have been selected, two residential areas were then selected in each district. Volunteers from any households living in the residential area with age group ranging from 18 to 60 years old regardless of their gender is the inclusion criteria for this study. Anyone who rejected to be included in the study and anyone who refuse or do not provide consent form were excluded. However, Subjects are free to not participate at any time before, during or after the sampling. The number of samples required was calculated using ‘Power and Sample Size Calculation’ software version 3.0.43 based on the formula stated by Fleiss JL [4].

## Ethics Statements

The ethics approval for this study was obtained from the Medical Research Ethics Committee, Ministry of Health [NMRR-19-3110-49409(IIR)] and from the Universiti Malaysia Sabah Research and Ethics Committee [JKEtika1/19(15)]. Informed consent was obtained from all participant in this study. The authors kept the ethical concerns into consideration when gathering data and ensured that the information obtained from the respondents were only utilized for research purposes.

## CRediT authorship contribution statement

**Noor Ain Haron:** Data curation, Writing – original draft. **Syed Sharizman Syed Abdul Rahim:** Software, Validation. **Jaeyres Jani:** Formal analysis. **Nur Athirah Yusof:** Validation. **Zarina Amin:** Visualization, Writing – review & editing. **Hooi Yuen Khoo:** Investigation. **Hai Yen Lee:** Investigation. **Chee Sieng Khor:** Methodology. **Kim Kee Tan:** Project administration. **Mohd Rohaizat Hassan:** Project administration. **Chin Mun Wong:** Supervision. **Hani Kartini Agustar:** Methodology. **Adia Aqilla Samsusah:** Conceptualization. **Rozita Hod:** Validation, Funding acquisition. **Sazaly Abu Bakar:** Supervision.

## Declaration of Competing Interest

The authors have no known competing financial interests or personal relationships that could have affected the work presented by the paper.

## References

[1] World Health Organization (WHO). (2016). https://apps.who.int/iris/handle/10665/204689.

[2] Andrade C., Menon V., Ameen S., Praharaj S.K. (2020). Designing and conducting knowledge, attitude, and practice surveys in psychiatry: practical guidance. Indian J. Psychol. Med..

[3] Puerta P., Ciannelli L., Johnson B. (2019). A simulation framework for evaluating multi-stage sampling designs in populations with spatially structured traits. Peer J..

[4] Fleiss J.L., Levin B., Paik M.C. (2013).

